# P-1422. Immunogenicity and Reactogenicity of Adjuvanted (Arexvy) and Non-Adjuvanted (Abrysvo) RSV Vaccines in Immunocompromised Individuals with Hematological Malignancies

**DOI:** 10.1093/ofid/ofaf695.1609

**Published:** 2026-01-11

**Authors:** Alisse Hannaford, Natalie E Izaguirre, Zoe Swank, Simon D Van Haren, Bridget Yates, Urwah Kanwal, Hannah Levine, Alexandra Tong, Maria Shehata, Fathia Oladipupo, Kevin Ryff, Xiaofang Li, Aidan Eustace, Lindsey Parisi, Louise L Hansen, Colleen J Sedney, Sanya Thomas, David Walt, Ofer Levy, Amar H Kelkar, Robert Soiffer, Vincent Ho, Zhou Lan, Lindsey R Baden, Nicolas C Issa, Ann E Woolley, Amy C Sherman

**Affiliations:** Brigham and Women's Hospital, Boston, MA; Brigham and Women's Hospital, Boston, MA; Brigham and Women's Hospital, Boston, MA; Harvard Medical School, Boston, Massachusetts; Brigham and Women's Hospital, Boston, MA; Brigham and Women's Hospital, Boston, MA; Brigham & Women's Hospital, Boston, Massachusetts; Brigham and Women's Hospital, Boston, MA; Brigham and Women's Hospital, Boston, MA; Brigham and Women's Hospital, Boston, MA; Brigham and Women's Hospital, Boston, MA; Brigham and Women's Hospital, Boston, MA; Brigham and Women's Hospital, Boston, MA; Brigham and Women's Hospital, Boston, MA; Brigham and Women's Hospital, Boston, MA; Boston Children's Hospital, Boston, Massachusetts; Boston Children's Hospital/Harvard Medical School, Boston, Massachusetts; Harvard Medical School/Brigham and Women's Hospital/Wyss Institute, Boston, Massachusetts; Boston Children's Hospital/Harvard Medical School/Broad Institute of MIT and Harvard, Boston, Massachusetts; Dana-Farber Cancer Institute / Harvard Medical School, Boston, Massachusetts; Brigham and Women's Hospital, Boston, MA; Brigham and Women's Hospital, Boston, MA; Brigham and Women's Hospital, Boston, MA; Brigham and Women's Hospital, Boston, MA; Brigham & Women's Hospital, Boston, Massachusetts; Brigham and Women's Hospital, Boston, MA; Brigham and Women's Hospital, Boston, MA

## Abstract

**Background:**

Given the limited data regarding RSV vaccine immunogenicity in individuals with hematological malignancies (HM), we evaluated the immune response of the RSV adjuvanted (*Arexvy*, GSK) and non-adjuvanted (*Abrysvo*, Pfizer) vaccines in people with HM as compared to healthy controls (HC).

**Table 1:**
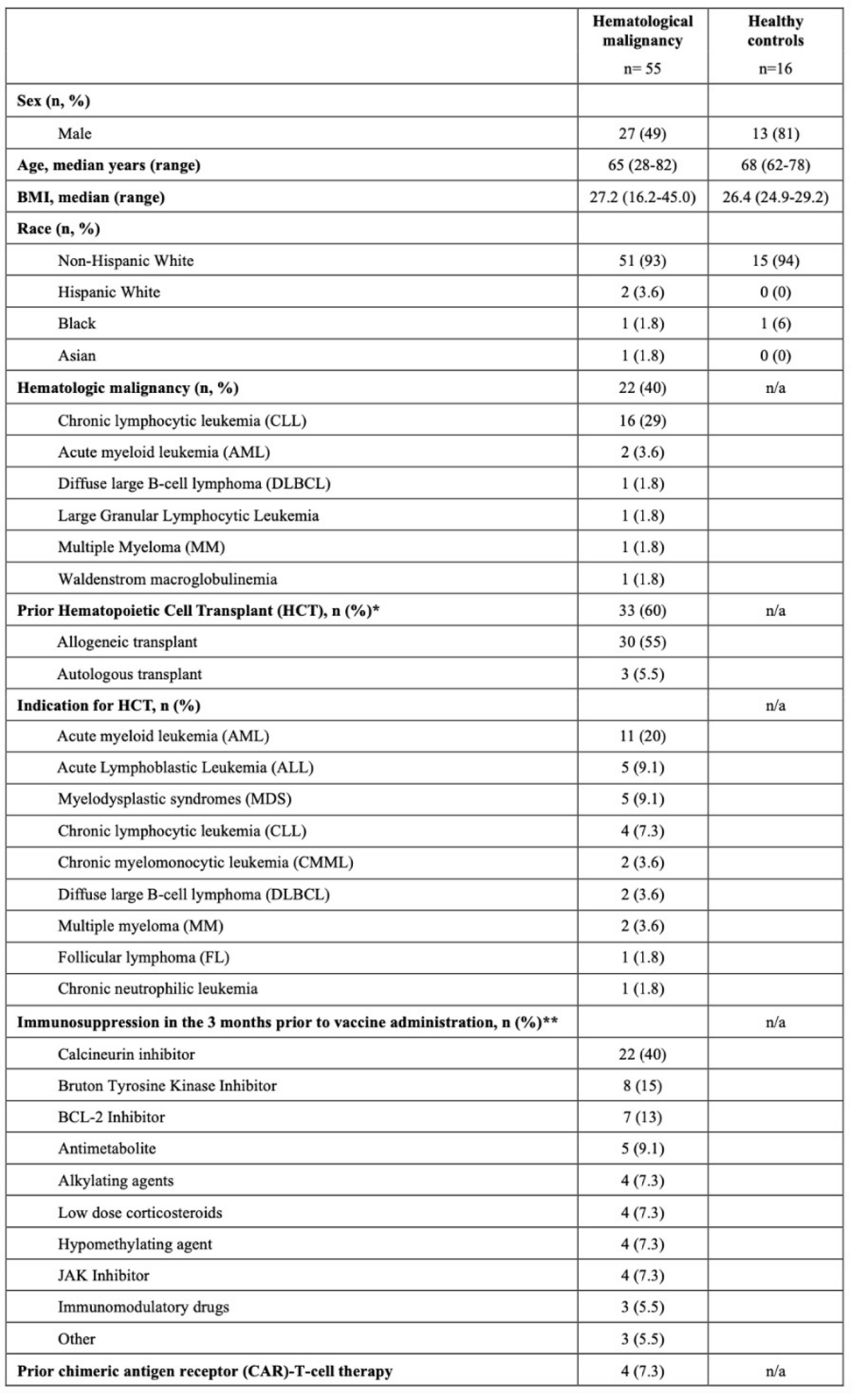
Baseline participant characteristics including demographic information, underlying hematologic malignancy, history of prior hematopoietic cell transplantation (HCT), indication for HCT, and immunosuppression used in the three months prior to vaccine administration. *Prior HCT recipients were eligible to receive vaccination if they were >100 days from transplant. **Participants could be on more than one immunosuppressive agent.

**Table 2:**
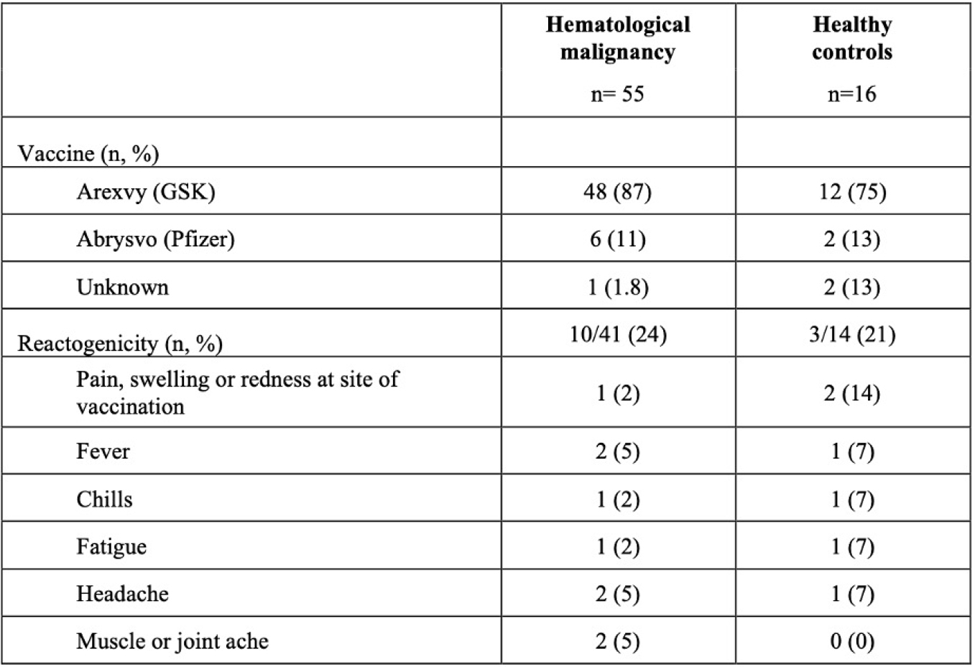
Vaccine type received and reactogenicity symptoms documented in the first 72 hours post-vaccination.

**Methods:**

A prospective, single-center study enrolled individuals with HM and healthy adults eligible for RSV vaccination. Baseline characteristics, malignancy diagnosis, and therapies were collected. Reactogenicity symptoms were recorded after vaccination. Blood was collected at baseline, 1, 3 and 6 months post-vaccination to measure anti-fusion glycoprotein (anti-FGP) antibodies (Abs) to RSV A and B (vaccine response), anti-nucleoprotein (anti-N) Abs (natural infection) and RSV-specific CD4^+^ and CD8^+^ T cells. The Mann-Whitney test compared the magnitude of response (GraphPad Prism 10.3.1).

**Figure 1:**
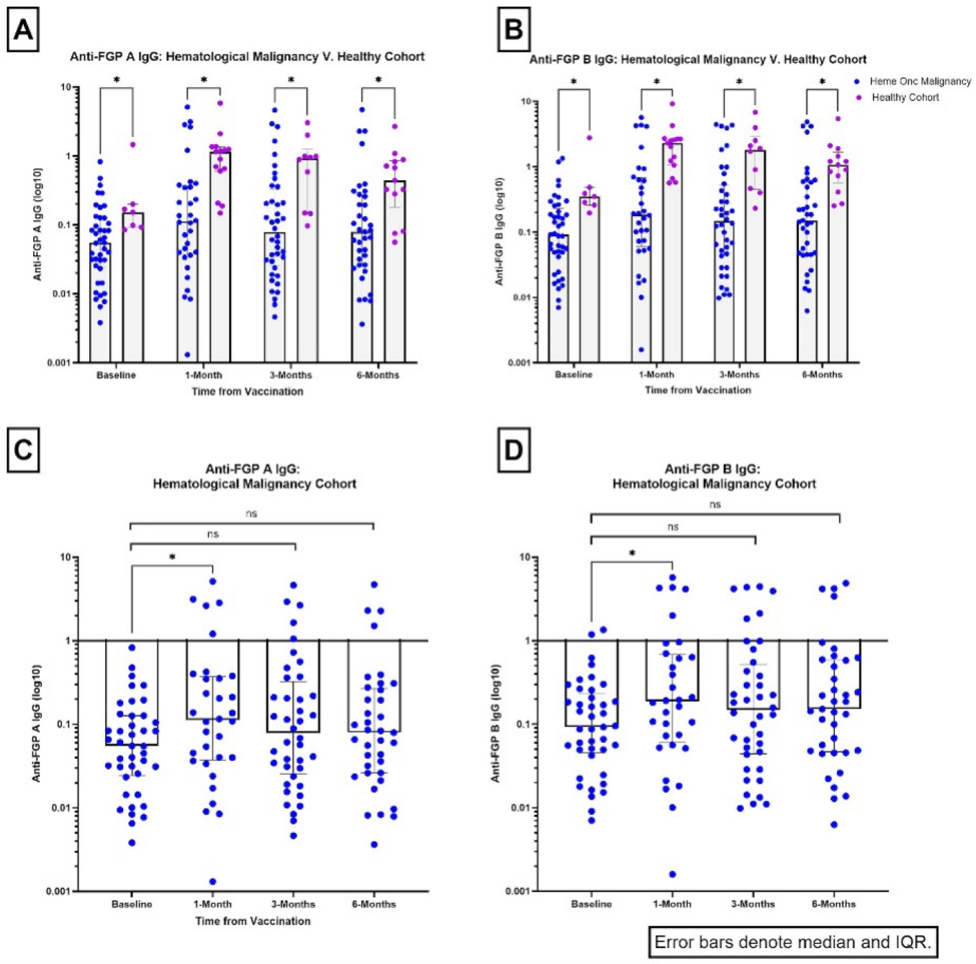
The anti-fusion glycoprotein (FGP) titers against RSV A (panel A) and RSV B (panel B), measured via the Single Molecular Array (Simoa) assay, are shown for the hematological malignancy cohort, denoted by the blue dots, and the healthy control cohort, denoted by the purple dots. Statistical significance was set at a level of alpha =0.05. Participants in the healthy cohort had significantly higher anti-FGP A and B titers as compared to participants in the hematological malignancy cohort, for all timepoints. Panels C and D show anti-FGP A and B IgG titers, respectively, for the hematological malignancy cohort only. There was a significant increase in both anti-FGP A and B titers at 1 month after vaccination, indicating response to the vaccine. However, when the 3-month and 6-month titers were compared to baseline titers, there was no significant difference, indicating a lack of a durable response to vaccination.

**Figure 2:**
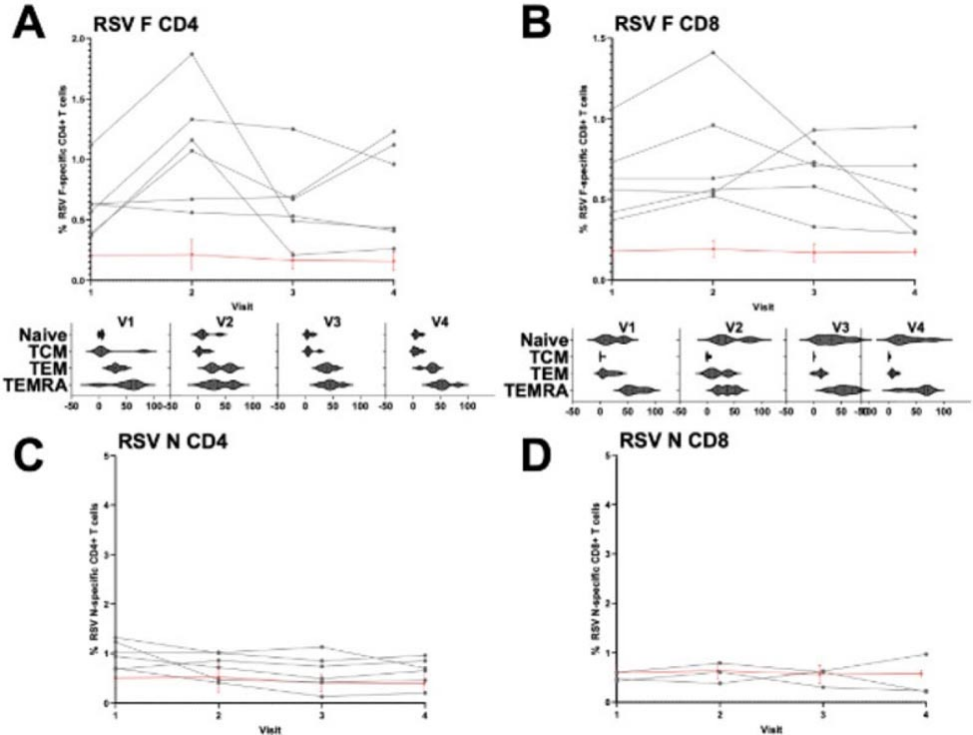
Cell-mediated immunity was quantified in a subset of participants (n=6) using an activation-induced marker (AIM) assay with spectral flow cytometry to determine peptide-pool-reactive T cells. Vaccine-induced CD4+ and CD8+ T cell responses (F protein, panels A and B) were quantified, with phenotypic analysis shown for V1-V4 as frequency of naive, central memory (TCM), effector memory (TEM), and effector memory T cells re-expressing CD45RA (TEMRA). Community viral exposure-induced CD4+ and CD8+ T cells (N, panels C and D) are shown over time by connecting lines for each participant. DMSO-controls are shown as aggregate with SEM (red line). Visit 1 (baseline), Visit 2 (1-month), Visit 3 (3-months) and Visit 4 (6-months) post-vaccination.

**Results:**

Between Aug 2023 and Mar 2025, we enrolled HM (N=55) and HC (N=16) cohorts. Demographic and treatment characteristics are described in Table 1. One quarter of each cohort experienced mild reactogenicity symptoms (Table 2). Anti-FGP IgG Abs against A and B were significantly higher in HCs as compared to the HM cohort for all time points (Fig. 1A/B). HM participants had a significant increase in titers at 1-month post-vaccination (Fig. 1C/D, Anti-FGP A: baseline median 0.055, 1-month median 0.11; p=0.03; Anti-FGP B: baseline median 0.092, 1-month median 0.19; p=0.04) although did not have a durable response at 3 or 6 months. Overall, there were no significant differences between baseline anti-N titers and the other three time points for either cohort except for one participant who was diagnosed with RSV infection. There was modest induction of RSV F-specific CD4^+^ T cells (∼1-2% of total CD4+ T cells) in a subset of HM participants, but minimal or absent induction of F-specific CD8^+^ T cells.

**Conclusion:**

RSV vaccination elicited both humoral and cellular responses in HM participants at 1 month, though responses were reduced as compared to HCs with poor durability. We will next evaluate predictors of immunogenicity to improve vaccine strategies for this vulnerable population.

**Disclosures:**

David Walt, PhD, Quanterix Corporation: Ownership Interest|Simoa technology: Board Member|Simoa technology: Ownership Interest Lindsey R. Baden, MD, Bill & Melinda Gates Foundation: Grant/Research Support|Bill & Melinda Gates Foundation: Collaboration of clinical trials conducted|COVID Vaccine Prevention Network: Collaboration of clinical trials conducted|Food and Drug Administration: Advisor/Consultant|HIV Vaccine Trials Network: Collaboration of clinical trials conducted|International AIDS Vaccine Initiative: Collaboration of clinical trials conducted|Johnson & Johnson: Collaboration of clinical trials conducted|Military HIV Research Program: Collaboration of clinical trials conducted|Moderna, Inc.: Collaboration of clinical trials conducted|National Institutes of Health: Advisor/Consultant|National Institutes of Health: Grant/Research Support|National Institutes of Health: Collaboration of clinical trials conducted|The Ragon Institute: Collaboration of clinical trials conducted|Wellcome Trust: Grant/Research Support Nicolas C. Issa, MD, AiCuris: Grant/Research Support|Astellas: Grant/Research Support|Boehringer Inglheim: Advisor/Consultant|Fujifilm: Grant/Research Support|GSK: Grant/Research Support|Merck: Grant/Research Support|Moderna: Grant/Research Support

